# Enhanced Oral Delivery of Docetaxel Using Thiolated Chitosan Nanoparticles: Preparation, In Vitro and In Vivo Studies

**DOI:** 10.1155/2013/150478

**Published:** 2013-07-21

**Authors:** Shahrooz Saremi, Rassoul Dinarvand, Abbas Kebriaeezadeh, Seyed Nasser Ostad, Fatemeh Atyabi

**Affiliations:** ^1^Department of Pharmaceutics, Faculty of Pharmacy, Tehran University of Medical Sciences, Tehran 1417614411, Iran; ^2^R&D Department, Osvah Pharmaceutical Co., Tehran, Iran; ^3^Nanotechnology Research Centre, Tehran University of Medical Sciences, Tehran 1417614411, Iran; ^4^Department of Toxicology and Pharmacology, Faculty of Pharmacy, Tehran University of Medical Sciences, Tehran, Iran

## Abstract

The aim of this study was to evaluate a nanoparticulate system with mucoadhesion properties composed of a core of polymethyl methacrylate surrounded by a shell of thiolated chitosan (Ch-GSH-pMMA) for enhancing oral bioavailability of docetaxel (DTX), an anticancer drug. DTX-loaded nanoparticles were prepared by emulsion polymerization method using cerium ammonium nitrate as an initiator. Physicochemical properties of the nanoparticles such as particle size, size distribution, morphology, drug loading, and entrapment efficiency were characterized. The pharmacokinetic study was carried out in vivo using wistar rats. The half-life of DTX-loaded NPs was about 9 times longer than oral DTX used as positive control. The oral bioavailability of DTX was increased to 68.9% for DTX-loaded nanoparticles compared to 6.5% for positive control. The nanoparticles showed stronger effect on the reduction of the transepithelial electrical resistance (TEER) of Caco-2 cell monolayer by opening the tight junctions. According to apparent permeability coefficient (*P*_app_) results, the DTX-loaded NPs showed more specific permeation across the Caco-2 cell monolayer in comparison to the DTX. In conclusion, the nanoparticles prepared in this study showed promising results for the development of an oral drug delivery system for anticancer drugs.

## 1. Introduction

In recent years, many works have been focused on the development of oral chemotherapy systems. Docetaxel (DTX) is regarded as one of the most effective drugs used in chemotherapy. DTX is a semisynthetic taxoid extract from *Taxus baccata* (European yew tree) and is used as antineoplastic agent against breast, ovarian, lung, head, and neck cancers [1–3]. The mechanism of action of DTX like other taxanes is inhibiting microtubule depolymerization [4, 5]. Because of stronger binding of DTX to tubulin it shows about 2–4 times more cytotoxicity effects on tumor cells than that of paclitaxel [6]. However DTX like other taxans has a poor oral absorption from gastrointestinal (GI) tract [7] and the only dosage form of DTX in the market is an injection dosage form (Taxotere). The main reasons for the poor oral bioavailability of DTX are related to low solubility of DTX in water, its high affinity to the multidrug efflux pump P-glycoprotein (P-gp), and hepatic first pass metabolism [8, 9]. Due to the poor solubility of DTX in water, it has been formulated as a solution using high amount of Tween 80 in ethanol (50 : 50 v/v). High concentration of solubilizers in its formulation causes toxic effects and allergic reactions [10]. Various methods have been suggested to overcome these problems such as applying a P-gp/P450 inhibitor such as cyclosporine A [11, 12], formulated as liposomes [13, 14], emulsions [15, 16], polymeric nanoparticles [17–21], and conjugation of DTX with water soluble polymers [22, 23].

Preparing surface modified polymeric nanoparticle may also be regarded as an effective mode of overcoming these problems. Nanoparticles, due to their unique properties and surface characteristics, can protect the drug from P-gp, cytochrome P-450, and from the destructive factors in the GI tract and can increase the permeability of drugs through the gastrointestinal barrier [24, 25]. Dong and Feng [19] developed nanoparticles using biodegradable polymers and showed that these polymeric nanoparticles can significantly increase oral bioavailability of DTX in rat. In another study, Peltier et al. [26] reported an increase in transport through the intestinal barrier and oral bioavailability of paclitaxel by lipid nanoparticles. Agüeros et al. [27] showed that, when paclitaxel was encapsulated in a complex of cyclodextrins and poly (anhydride) nanoparticles, its bioavailability was significantly increased. These reports confirm that nanoparticulate systems with unique properties can increase the transport of poorly water-soluble compounds across the GI barrier. In this study we investigated the capacity of prepared thiolated nanoparticles based on thiolated chitosan to improve the oral bioavailability of DTX as a model anticancer drug with poor oral absorption. Roldo et al. [28] showed that the mucoadhesive properties of chitosan was enhanced 140-fold due to the immobilization of thiol groups on the polymer. Formation of disulfide bonds between the thiolated polymer and cysteine-rich subdomains of the mucus gel layer is responsible for this improvement [29]. There are many reports on the application of thiolated chitosan for enhancing permeability, mucoadhesivity and intestinal absorption of active agents [30–33].

Recently, we reported that DTX and paclitaxel could be easily entrapped in thiolated chitosan-pMMA nanoparticles [34, 35]. It was shown that drug-loaded NPs increased the cytotoxicity of DTX and transportation of DTX across the jejunum of rats was facilitated in ex vivo study. TEER value of Caco-2 cell monolayer was also measured to evaluate the influence of the thiolated nanoparticles on the quality of intestinal tight junctions in male Wistar rats.

## 2. Materials and Methods

### 2.1. Materials

Docetaxel was obtained from Cipla (Mumbai, India), Taxotere, an injectable commercially available formulation of DTX, was from Sanofi-Aventis (France), and Chitosan (ChitoClear) with molecular weight of 20 and 50 kDa and degree of deacetylation of about 89% was purchased from Primex (Karmoy, Norway). L-Glutathione reduced form (GSH), 1-ethyl-3-(3-dimethylaminopropyl) carbodiimide hydrochloride (EDC), N-hydroxysuccinimide (NHS), methyl methacrylate (MMA), ammonium cerium nitrate, sodium nitrite, hydrochloric acid, glacial acetic acid, sodium hydroxide, and potassium hydrogen phosphate were all purchased from Merck (Darmstadt, Germany). Ellman's reagents, 5, 50-dithiobis (2-nitro benzoic acid), were obtained from Sigma (St. Louis, MO, USA). Caco-2 cell lines were obtained from Pasteur Institute (Tehran, Iran). 3-(4,5-Dimethylthiazol-2-yl)-2,5-diphenyl tetrazolium bromide) (MTT) and all of the cell culture mediums were purchased from Sigma-Aldrich (St. Louis, MO, USA). All other chemicals were of analytical grade.

### 2.2. Preparation of DTX-Loaded Nanoparticle

Thiolated chitosan was prepared with covalent attachment of reduced glutathione to chitosan in the presence of EDC and NHS according to method described in our previous study [36]. The DTX-loaded nanoparticles were prepared by using a modified radical polymerization method [37]. Conjugated chitosan (37.5 mg) was dissolved in 4 mL nitric acid (0.2 M) in a two-necked flask under gentle stirring and nitrogen bubbling at 40°C. After 10 min, under vigorous magnetic stirring, solution of 0.08 M cerium IV ammonium nitrate (CAN) in 0.2 M nitric acid was added to obtain a 5 mL solution. DTX was dissolved in a 0.5 mL of methanol under stirring. Then 0.25 mL MMA was added to obtain a clear solution. The added amount of DTX was 10.90 mg or 4% (w/w) based on total weight of polymers (MMA and thiolated chitosan). Nitrogen bubbling was kept for additional 10 min. The reaction was allowed to continue at 40°C under gentle stirring for 40 min. The reaction was left to reach room temperature, and the pH of obtained suspension adjusted to 4.5 by addition of sodium hydroxide (1 N) dropwise. Then nanoparticles suspension was purified by dialyzing against acetic acid solution, used for the removal of the remained methacrylic acid monomers, (1 L, 16 *μ*mol/L) in demineralized water for 90 min twice and once overnight using Sigma dialysis tubes Mw cutoff of 12 kDa. The frozen samples were lyophilized at −50°C and 0.01 mbar and stored at 4°C until further use.

### 2.3. Characterization of Nanoparticles

The mean diameter and size distribution of nanoparticles were determined by dynamic light scattering using Zetasizer (Nano-ZS, Malvern, Worcestershire, UK) at wavelength of 633 nm at 25°C with an angle detection of 90°. The samples were diluted in acetic acid (16 *μ*mol/L) in deionized water, and three subsequent measurements were determined for each sample, and the result was expressed as mean size ± S.D. The zeta potential measurements were performed by laser Doppler electrophoresis using Zetasizer (Nano-ZS, Malvern, Worcestershire, UK). In order to maintain a constant ionic strength, the samples were diluted (1 : 50 v/v) in NaCl 1 mM (pH_6.5) [38]. Each sample was repeatedly measured three times.

The surface morphology of nanoparticles was evaluated by using a scanning electron microscope (XL 30, Philips, Eindhoven, the Netherlands). Nanoparticle suspensions were successively diluted in deionized water to 1/50 (v/v). The dilutions were spread on an aluminum disc and dried at room temperature before the analysis. The dried nanoparticles were then coated with a thin layer of gold metal using a sputter coater (SCD 005, Bal-Tec, Switzerland).

### 2.4. Drug Loading and Entrapment Efficiency

The entrapment efficiency (EE) of the process was determined indirectly upon separation of the drug-loaded NPs (after dialysis) by ultracentrifugation at 25,000 rpm, 8°C for 30 minutes from the aqueous medium containing free DTX. The amount of free DTX in the supernatant was measured using HPLC. Isocratic reversed-phase HPLC was performed using a Knauer HPLC system (Knauer, Berlin, Germany) with a 5 *μ* Bondapak C18 column (Waters, Milford, MA, USA). The mobile phase consisted of 75 : 25 (v/v) methanol/water and was delivered at a flow rate of 1.0 mL/min. Eluted compounds were detected at 227 nm using a Spectra100 UV-Vis detector. The standard curve was found to be linear in the concentration range 0.5 *μ*g/mL–50 *μ*g/mL with *R* ^2^ = 0.9999.

The EE of DTX NPs was calculated as the ratio of DTX loaded into the NPs with respect to the total amount of DTX used for preparation of the original mixture as follows:

(1) $$\begin{array}{c} \text{EE}\left(\%\right)=\frac{\left(\text{DTXt}-\text{DTXf}\right)}{\text{DTXt}}\times 100, \end{array}$$

where DTXt is the total amount of DTX used for preparation of the original mixture and DTXf is the free DTX amount recovered in the supernatant. All samples were measured in triplicate. Drug loading was calculated as follows [39]:

(2) $$\begin{array}{c} \text{DL}\left(\%\right)=\left(\frac{\text{Weight}\;\text{of}\;\text{loaded}\;\text{drug}}{\text{Weight}\;\text{of}\;\text{NPs}}\right)\times 100. \end{array}$$

### 2.5. In Vitro Drug Release Study

Drug release from DTX-loaded nanoparticles was studied by incubating the nanoparticles in phosphate buffer solutions (PBS), at pH 7.4, at 37°C. Two mg of nanoparticles were dispersed in 5 mL of release medium (PBS of pH 7.4 containing 0.1% w/v Tween 80) in a dialysis tube (Sigma dialysis tubes Mw cutoff 12 kDa), and the closed dialysis bag immersed in 20 mL release medium in a centrifuge tube. Tween 80 was used to increase the solubility of DTX in the buffer solution to maintain sink condition. The tube was placed in a shaker bath at 37°C and shaken horizontally at 100 cycles/min. At given time intervals, 15 mL of samples were withdrawn and replaced with the same volume of fresh medium. The samples were filtered through a 0.22 *μ*m filter and were analyzed for the amount of DTX using HPLC.

### 2.6. Caco-2 Cell Culture Study

Caco-2 cells, with a passage number 40–45, were cultured on polycarbonate membrane filters (pore size 0.4 *μ*m, area 0.47 cm^2^) in 24-well plates (Nunc, Roskilde, Denmark) at a seeding density of 4 × 10^5^ cells/cm^2^. The RPMI 1640 (50% v/v), Dulbecco's modified Eagle's medium (35% v/v DMEM, Sigma, pH 7.4), with 4500 mg/L glucose and 15% fetal bovine serum (FBS) containing 1% penicillin-streptomycin was used as medium for cell culture. The culture medium was added to both the apical (300 *μ*L) and basolateral (700 *μ*L) of filter insert and was changed every other day for the first 10 days and every day thereafter until 21 days. The cells were left at 37°C in an incubator under atmosphere of 95% air and 5% CO_2_ at 90% relative humidity. One hour before the experiments, the medium was changed to the transport buffer containing: Hank's balanced salt solution (HBSS) buffered with 30 mM n-(2-hydroxyethyl) piperazine-n-(2-ethanesulfonic acid) (HEPES) at pH 5.5 and the cells were allowed to equilibrate for 1 h.

### 2.7. Determination of the Transepithelial Electrical Resistance (TEER)

The integrity of cell monolayer on the filters was examined by measuring the transepithelial electrical resistance (TEER) using an EVOM2 instrument (World precision Instruments, Sarasota, FL) connected to a pair of chopstick electrodes. TEER test was carried out to examine the ability of DTX and DTX-loaded NPs on the opening of the tight junctions at predetermined time intervals of 0, 0.5, 1, 2, 3, 4, and 24 h. The experiments were done in triplicate.

### 2.8. Permeation Study

Permeation of samples was determined as described by Sadeghi et al. [40] with some modifications. Transport of different dispersions of free DTX, Taxotere, commercially available formulation of injectable docetaxel (F-DTX), and Ch20-GSH-DTX and Ch50-GSH-DTX NPs was studied from the apical to basolateral direction on Caco-2 cells. The test solutions were produced by diluting with transition buffer (HEPES-HPSS) at 2 *μ*M DTX concentrations. The upper chamber (apical side) was filled with 300 *μ*L of the different test solutions and the lower chamber (basolateral side) was filled with 700 *μ*L of the growth medium followed by incubation at 37°C with 5% CO_2_/95% air. At predetermined time of 30, 60, 120, and 240 min 300 *μ*L samples were withdrawn from the basolateral side of filter and replaced with equal volumes of fresh HBSS-HEPES. The samples were analyzed for the DTX content using the HPLC method. After four hours and completion of the permeability studies, the samples were carefully removed from the apical part and the cell monolayer was rinsed with HBSS-HEPES, and the medium was then replaced with fresh culture medium. The monolayer was incubated for 24 h at 37°C in regular cell culture conditions. The TEER was monitored during the experiment and at 24 h. Results were corrected for dilution and expressed as cumulative transport with time. All the experiments were done in triplicate.

Apparent permeability coefficients (*P*_app_) were calculated using the following equation:

(3) $$\begin{array}{c} P_{\text{app}}=\frac{Q}{{AC}_{0}t}, \end{array}$$

where *P*_app_ is the apparent permeability coefficient (cm/s), *Q* is the total amount permeated throughout the incubation time (*μ*g), *A* is the diffusion area of the monolayer (cm^2^), *C*_0_ is the initial concentration of the DTX in the apical part (*μ*g/cm^3^), and *t* is the total time of the experiments.

### 2.9. In Vivo Study

Male wistar rats of 250–280 g and 10–12 weeks old (provided by Pasteur Institute of Iran) were kept at temperature of 25 ± 2°C and relative humidity of 50–60% under natural light/dark conditions for one week before experiments. The animals were distributed into three groups. Group 1 received an i.v. injection of F-DTX (*n* = 5). Groups 2 and 3, used for oral administration of DTX, were allowed to fast and unlimited for water accessibility for 12 h followed by receiving an oral delivery of F-DTX and DTX-loaded NPs (*n* = 5), respectively. The study protocol was approved by the Institutional Review Board of Pharmaceutical Research Centre of Tehran University of Medical Sciences.

The NP formulation was dispersed in, and F-DTX was diluted with saline and was orally administered at the same DTX dose of 10 mg/kg body weight. For Groups 1 and 2, blood samples were collected at 0, 0.5, 1, 2, 3, 5, 8, 12, 24, and 48 h. For Group 3, blood samples were collected at 0, 0.5, 1, 2, 3, 5, 8, 12, 24, 48, 72, 120, 168, 196, 216, 240, 360, and 480 h after administration. Plasma samples were harvested by centrifugation at 14,000 rpm for 15 min and stored at −40°C for HPLC analysis. 

### 2.10. Drug Loading and Release Measurements

HPLC method as reported previously [34] was used for the analysis of DTX for the drug content, transport, and in vitro release studies. Samples were directly injected (20 *μ*L) into the HPLC system without further treatment, while plasma samples were extracted with chloroform and dichloromethane before injection. Briefly, 500 *μ*L of plasma was spiked with 200 *μ*L of phosphate buffer (pH 6.5) and 25 *μ*L of paclitaxel (20 *μ*g/mL in ethanol) as the internal standard. DTX was extracted with 5 mL chloroform and 700 *μ*L dichloromethane by vigorous mixing for 1 min. After centrifugation at 3500 rpm for 15 min, the organic phase was collected. The organic phase was dried under nitrogen gas stream at 40°C. The residue was then dissolved with 70 *μ*L of mobile phase and mixed for 5 min. The solution was centrifuged for 2 min at 3000 rpm, and 20 *μ*L of the supernatant was injected into the HPLC system (Knauer, Berlin, Germany) using a spectra 100 UV-Vis detector. 

For plasma samples a Nucleodur C18 Gravity HPLC packed column (4.6 mm × 250 mm, 5 *μ*m, Macherey-Nagel, Germany) was used at room temperature. The mobile phase (phosphate buffer (pH 6.0): methanol (70 : 30 v/v)) flowed at rate of 1.3 mL/min. Eluted compounds were detected at 227 nm. The total run time was 25 min.

### 2.11. Statistical Analysis

Data are reported as mean ± standard deviation from 3 independent experiments. Statistical significance between mean values was calculated using independent sample *t*-test and one-way analysis of variance (ANOVA). Probability values <0.05 were considered significant.

## 3. Results and Discussions

### 3.1. Preparation and Characterization of DTX-Loaded-Nanoparticles

Table 1 shows the particle size, polydispersity index (PDI), and zeta potential of NPs prepared using thiolated chitosan having two different molecular weight of 20 and 50 kDa (Ch20-GSH, Ch50-GSH). As can be seen the mean diameters of the NPs were 198 nm for Ch20 and 262 nm for Ch50. As expected nanoparticles prepared with higher molecular weight chitosan were bigger in size compared with those prepared with smaller molecular weight chitosan. This may be due to the higher viscosity of polymeric droplets of higher molecular weight chitosan compared with those of smaller molecular weight chitosan. The results were very similar to the results obtained by Bernkop-Schnürch et al. [39]. The PDI values of NPs were 0.12 and 0.20 for Ch20 and Ch50, respectively, indicating a more homogenous size distribution for nanoparticles prepared by lower molecular weight chitosan. The zeta potential of the NPs was positive due to the presence of amino groups of chitosan around the core of pMMA. This positive zeta potential and the existing of thiol groups on the surface of particles are favorable for making strong electrostatic and disulfide bonds with negatively charged mucosa which can prevent elimination of the nanoparticles through the alimentary canal [41]. 

**Figure 1:**
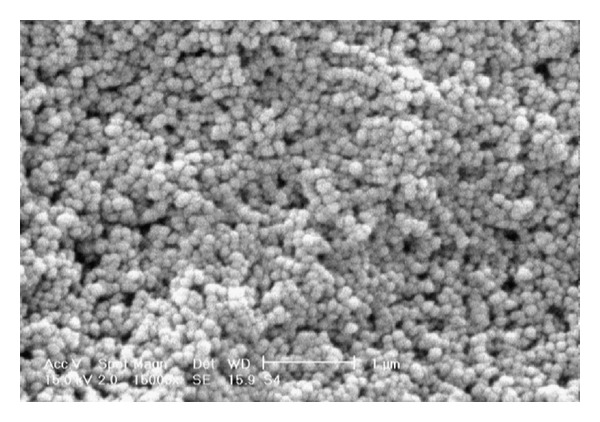
Scanning electron micrograph of DTX-loaded nanoparticles (magnification of 150,000x).

Figure 1 shows the SEM image of the NPs that are spherical and uniform.

### 3.2. Entrapment Efficiency and Drug Loading of NPs

Table 1 shows the EE and DL of the NPs. As can be seen in this table, the EE of Ch20-GSH and Ch50-GSH NPs is 93.5% and 89.2%, respectively. This high EE might be due to the tendency of DTX as a hydrophobic molecule to enter the hydrophobic core (pMMA) of the NPs. Ideally, a successful nanoparticulate system should have a high DL capacity. The DL of Ch20-GSH and Ch50-GSH NPs was 18.4% and 18.2%, respectively. A high DL capacity was expected, due to the hydrophobicity property of DTX.

### 3.3. In Vitro Drug Release Study

**Figure 2:**
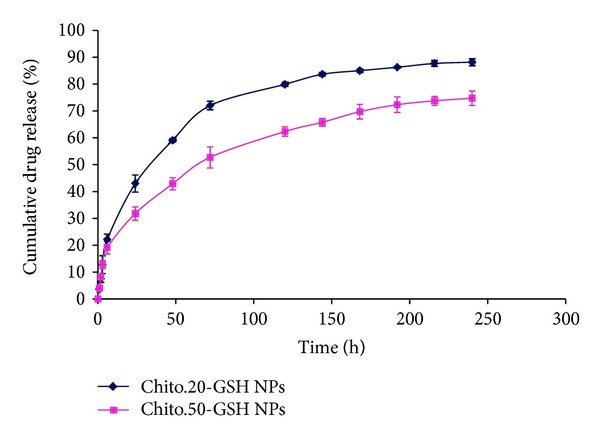
In vitro drug release profile of the Ch20-GSH (♦) and Ch50-GSH (■) NPs. Experiments were carried out in triplicate (*n* = 3).

Drug release study showed a sustained release profile as up to 80% of its drug content released during 10 days from Ch20 and about 70% from Ch50 NPs in phosphate buffer solution with pH of 7.4 (Figure 2). The hydrophobic core of nanoparticles acts as a barrier against the diffusion of entrapped DTX from the polymeric matrix into the aqueous solution resulting in a slow release of drugs, desirable for thiolated nanoparticles. When NPs were created with Ch50, it was shown that they are dispersed in buffer, but some aggregation and formation of a mass of large particles may be seen. Difference between drug releases behavior of two nanoparticles prepared with chitosan with two different molecular weights might be related to the size of them. NPs prepared with higher molecular weight have bigger size in medium and form a more viscose layer around the particles after hydration with water.

### 3.4. Effect of Nanoparticle Suspension on TEER of Caco-2 Cell Monolayer

**Figure 3:**
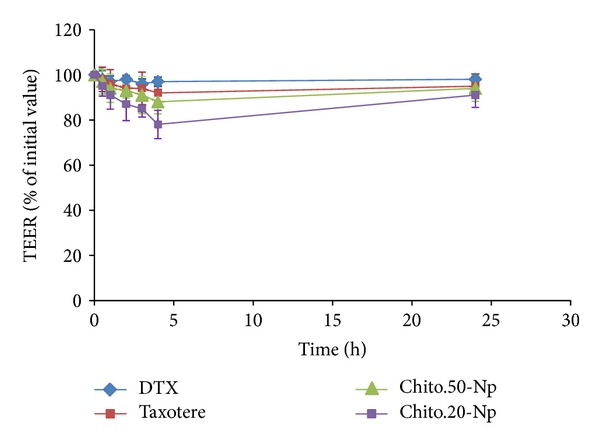
Effects of DTX and DTX-loaded nanoparticles on TEER of Caco-2 cell monolayer during the experiment and 24 h after rinsing the monolayer with HBSS-HEPES and applying culture medium on the monolayer. Data are expressed as means ± SD of three experiments.

The reversible effects of nanoparticles of thiolated chitosan on barrier properties and opening the intestinal tight junctions were studied by measuring the transepithelial electrical resistance (TEER) values across the Caco-2 cells. The results are presented as the percentage of the initial values at *t* = 0 min and are shown in Figure 3. As can be seen, the effect of nanoparticles on opening the tight junction is higher than that for free DTX or F-DTX. After four hours the quantity of opening tight junction with nanoparticles is about 80% for Ch20 and 88% for Ch50 of the initial value versus about 92% and 97% for F-DTX and free DTX, respectively. One of the possible mechanisms for uptake of nanoparticles via the intestinal tract is paracellular transport that is done through epithelial cells. In many studies it was demonstrated that nanoparticles based on chitosan are able to open tight junction and transport across the cell monolayer [40, 42, 43]. Chitosan derivatives can disrupt epithelial cell tight junctions and decrease the TEER value by two pathway: (1) interaction of their positive surface charge with the anionic components of the glycoprotein on the surface of the epithelial cells [25, 44] and (2) translocation of tight junction proteins from the plasma membrane where they are available to form tight junctions membrane to the cytoskeleton [45]. Nanoparticles prepared from smaller molecular weight chitosan (Ch-20) reduced the TEER value more substantially than higher molecular weight chitosan nanoparticles (Ch-50). TEER value of F-DTX was shown to be close to Ch-50 and much lower than free DTX. The reason for this observation may be related to the Tween 80 content of F-DTX. It has been shown that nonionic surfactants such as Tween 80 in a large dose are able to enhance the permeability of Caco-2 cell monolayer [46] and decrease the TEER value. F-DTX has a large volume of Tween 80 and can increase the permeability more than free DTX. The *P*_app_ values of the DTX in different formulations are shown in Table 2. The *P*_app_ value of DTX from Ch20-GSH, Ch50-GSH, F-DTX, and free DTX was 2.43, 2.14, 0.38 and 0.08, respectively. It showed that the apparent permeability values of nanoparticles were significantly higher than those from free DTX and F-DTX.

### 3.5. In Vivo Pharmacokinetics

**Figure 4:**
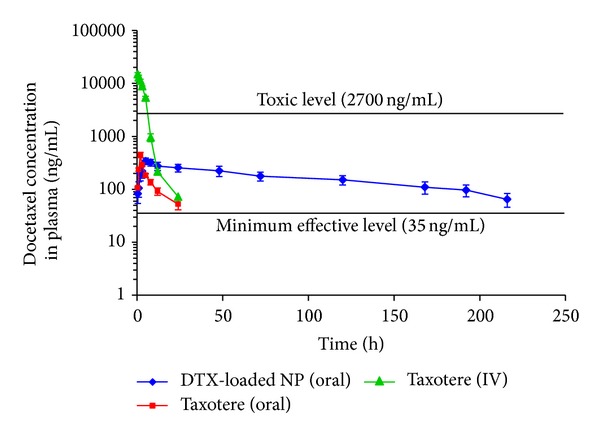
Plasma concentration-time profiles of docetaxel after bolus intravenous injection of F-DTX, oral administration of F-DTX or DTX-loaded NPs (equivalent to 10 mg/kg as docetaxel) to rats. Each data point represents the mean ± SD of five determinations.

Figure 4 shows the mean plasma concentration of DTX when administrated orally using F-DTX and DTX-loaded NPs compared to injected F-DTX at the same concentration (10 mg/kg) in Wistar rat animals (*n* = 5). Plasma level of DTX was measurable up to 216 h for NPs (p.o.) and 24 h for F-DTX when administered orally or intravenously. The most important pharmacokinetic parameters including *C*_max⁡_, *T*_max⁡_, *T*_1/2_, AUC_0−*∞*_, and MRT are summarized in Table 3. It can be seen that after intravenous administration of DTX, the drug plasma level reached to extremely high concentration value (14,744 ng/mL) above the maximum therapeutic level [47] which may cause serious side effects. Instead, oral F-DTX and oral NP formulation showed lower maximum drug concentrations that are in the therapeutic window (456 ng/mL and 341 ng/mL, resp.). As can be seen, drug half-life (*T*_1/2_) for oral administration of NPs was 102.5 h, that is, about 9-fold more than F-DTX when given orally. This may be due to the mucoadhesion of NPs that prolong their residence at the site of absorption. As expected the *T*_max⁡_ was increased to 5 h for Ch-GSH NPs, 2.5-fold of that for oral administration of F-DTX. Also the data illustrated that bioavailability of DTX formulated in Ch-GSH NPs is 68.9% which is about 10-fold more than that for oral bioavailability of F-DTX (6.5%). This significant increase in the oral bioavailability of DTX in the NPs formulation could be related to mucoadhesion properties, P-gp efflux inhibition, and permeability enhancing effects of thiolated chitosan. Given the prolonged plasma level of docetaxel when nanoparticles are given orally, the absorption of nanoparticles is a real possibility. Another explanation of this higher plasma level of docetaxel for nanoparticles may be related to the mucoadhesion of nanoparticles. In addition to that, it is well established that transmucosal transport of the P-gp substrates is strongly improved in the presence of thiolated chitosan. Glutathione and thiolated chitosan inhibit multidrug resistance P-glycoprotein activity in excised small intestine [46]. Therefore, when P-gp is inhibited, the bioavailability of substrates such as docetaxel is increased. Thiolated chitosan nanoparticles when administered orally could enhance oral bioavailability of DTX instead of current regimen of chemotherapy (IV injection). In addition, it can be regarded as a superior system when compared to other strategies that use P-gp/P450 inhibitors like cyclosporine-A with many side effects [11, 12].

## 4. Conclusion

In this study a core shell nanoparticulate system for the oral delivery of DTX with mucoadhesive properties for enhancing oral absorption of anticancer drugs is reported. Nanoparticles prepared in this study are superior to other nanoparticles such as PLGA NPs in terms of the following: (1) mucoadhesion property of thiolated chitosan provides better residence time of NPs in gastrointestinal tract, (2) achieving high drug entrapment efficiency, (3) surface hydrophilicity of chitosan NPs is favored compared to hydrophobic PLGA NPs, and (4) no hazardous organic solvent is used for the preparation of chitosan nanoparticles. Permeation study showed that nanoparticles could open tight junction of monolayer Caco-2 cells and increase paracellular transportation. In vivo experiment with Wistar rats showed a significant increase in the half-life of DTX in plasma in comparison to that of F-DTX after IV injection. One dose of oral nanoparticle formulation can release DTX as sustainable manner for 216 h in comparison of 24 h for oral administration of F-DTX at the same dose of 10 mg/kg of DTX. The oral bioavailability of Ch-GSH-PMMA NPs was about 10-fold higher than that of oral F-DTX.

## Figures and Tables

**Table 1:** Properties and characteristics of the Ch20- and Ch50-DTX-loaded NPs.

Formulation	DTX (mg)	Size (nm)	PDI	Zeta (mV)	DL (%)	EE (%)
Ch20-GSH-DTX 4%	10.90	198 ± 8.5	0.12	+31.6 ± 1.5	18.39	93.56
Ch50-GSH-DTX 4%	10.90	262 ± 7.0	0.23	+30.6 ± 4.4	18.22	89.22

**Table 2:** Apparent permeability (*P*_app_) of different samples of DTX: free DTX, F-DTX, and DTX-loaded NPs (*n* = 3; data are showed as mean ± SD); the difference *P* < 0.05 is considered as significant.

Test compound	Average *P*_app_* (×10^−6^ cm/s)
DTX	0.08 ± 0.14
F-DTX	0.38 ± 0.05
Ch50-GSH-DTX NPs	2.14 ± 0.22
Ch20-GSH-DTX NPs	2.43 ± 0.38

*P_app_: apparent permeability.

**Table 3:** Pharmacokinetics parameters in rats after i.v. administration of F-DTX and oral administration of F-DTX and Ch20-GSH-DTX NPs at the same 10 mg/kg drug dose.

PK Parameters	F-DTX (IV)	F-DTX (oral)	Ch20-GSH NPs (oral)
*T*_max⁡_ (h)	0.5	2	5
*C*_max⁡_ (ng/mL)	14,744 ± 2,354	456 ± 54.1	341 ± 47.5
AUC_0–*∞*_ (h·ng/mL)	65,245 ± 4,545	4,243 ± 207	44,998 ± 3,534
*T*_1/2_ (h)	2.7 ± 0.6	11.7 ± 1.45	102.5 ± 12.6
MRT (h)	3.2 ± 0.3	15.7 ± 1.6	144.0 ± 10.7
Absolute bioavailability	—	6.5%	—
Relative bioavailability	—	—	68.9%
